# Inflammatory cytokines and oral lichen planus: a Mendelian randomization study

**DOI:** 10.3389/fimmu.2024.1332317

**Published:** 2024-02-07

**Authors:** Xin Chen, Simin Zhang, Xiao Wu, Yuxi Lei, Bing Lei, Zhibai Zhao

**Affiliations:** ^1^ Department of Oral and Maxillofacial Surgery, Jiangyin People's Hospital Affiliated to Nantong University, Wuxi, Jiangsu, China; ^2^ Key Laboratory of Shaanxi Province for Craniofacial Precision Medicine Research, College of Stomatology, Xi’an Jiaotong University, Xi’an, Shaanxi, China; ^3^ Department of General Dentistry, College of Stomatology, Xi’an Jiaotong University, Xi’an, Shaanxi, China; ^4^ Endodontic Department, School of Stomatology, Nanjing Medical University, Nanjing, Jiangsu, China; ^5^ Department of Emergency Room, College of Stomatology, Xi’an Jiaotong University, Xi’an, Shaanxi, China

**Keywords:** Mendelian randomization, oral lichen planus, inflammatory cytokines, inflammation, immunity

## Abstract

**Background:**

Inflammatory cytokines have long been considered closely related to the development of oral lichen planus (OLP), and we further explored the causal relationship between the two by Mendelian randomization (MR) method.

**Methods:**

We performed bidirectional MR analyses by large genome-wide association studies (GWAS). The data included a large-scale OLP dataset, as well as datasets of 41 inflammatory cytokines. All data were obtained from the University of Bristol database, which includes 41 inflammatory cytokines, and the GWAS Catalog database, which includes 91 inflammatory cytokines. OLP data were obtained from the Finngen database, which includes 6411 cases and 405770 healthy controls. We used the inverse variance weighted (IVW) method, MR-Egger method, weighted median method, simple mode method and weighted mode method to analyze the causal relationship between inflammatory cytokines and OLP, and we also combined with sensitivity analysis to further verify the robustness of the results. We performed a meta-analysis of positive or potentially positive results for the same genes to confirm the reliability of the final results.

**Results:**

We primarily used the IVW analysis method, corrected using the Benjamin Hochberg (BH) method. When p<0.00038 (0.05/132), the results are significantly causal; when 0.00038<p<0.05, the results are potentially causal. We found a total of 7 inflammatory cytokines with significant or potential associations with OLP (University of Bristol database: 2, GWAS Catalog database: 5). In the reverse analysis, we found that a total of 30 inflammatory cytokines were significantly or potentially associated with OLP (University of Bristol database: 5, GWAS Catalog database: 25). After sensitivity analysis and meta-analysis, we finally determined that there was a causal relationship between a total of 3 inflammatory cytokines and OLP in the forward analysis, the most significant of which was FGF21 (p=0.02954, odds ratio (OR): 1.113, 95% confidence interval (95%CI): 1.011-1.226). In the reverse analysis, 14 inflammatory cytokines were causally associated with OLP, the most significant of which was PLAU (p=0.00002, OR: 0.951, 95%CI: 0.930-0.973).

**Conclusion:**

There is a causal association between OLP and some inflammatory cytokines, which may play an important role in the pathogenesis of OLP and require further attention.

## Introduction

Oral lichen planus (OLP) is a chronic inflammatory immune disease with a global prevalence of approximately 1% (1). A predominant phenotype of persistent and recurrent flare-ups characterizes it. Grayish-white pinhead-sized papular lesions in the form of lines, reticulations, or rings are the main clinical features, and in patients with more severe symptoms, congestion, blisters, or atrophy may also be present (2, 3). The World Health Organization (WHO) classifies OLP as an oral potentially malignant disease (OPMD), which can develop into oral squamous cell carcinoma (OSCC) in severe cases (1). The etiology of OLP is unclear, but immune system abnormalities are essential in developing the disease. Immune system abnormalities in OLP are reflected in the production of large amounts of inflammatory mediators in the lesion area and in the peripheral blood, which affects the interactions between keratinocytes and mononuclear cells (4, 5). Previous studies have found that many cytokines are present in these inflammatory mediators, suggesting that inflammatory cytokines play an essential role in the pathogenesis of OLP (6).

Cytokines are mainly composed of small peptide proteins that can be synthesized and secreted by immune cells and some non-immune cells, and are potent mediators in various physiological responses of the body. Most cytokines are pleiotropic and play different roles in different physiological environments. Cytokines have powerful immunomodulatory effects, and cytokine abnormalities may lead to immunodeficiency, allergic reactions, and autoimmune diseases (ADs) (6, 7). Previous studies have shown that OLP is closely associated with various inflammatory cytokines, and the abnormal expression of various inflammatory cytokines is prevalent in the lesion area, saliva, and peripheral blood mononuclear cells (PBMCs) of OLP patients (8, 9). In addition, genetic polymorphisms of inflammation-related factors, such as TNF-α, IL-4, and IL-17, have also been associated with susceptibility to OLP. Studies on the role of these cytokines in the pathogenesis of OLP will contribute to a deeper understanding of the pathogenesis of OLP and the exploration of new therapeutic approaches (10–12).

Mendelian randomization (MR) method is an emerging statistical method in statistics that uses genetic variation as the instrumental variable (IV) to detect and quantify causality. MR overcomes the effects of potential confounding and reverse cause by utilizing genotyped IVs and is based on the three main assumptions of “IVs are strongly associated with exposure factors”, “IVs are not associated with confounding factors” and “IVs are associated with outcomes only through exposure”, which makes the strength of the argument for associations more reliable than that of observational studies or even randomized controlled studies. MR Studies are based on one or more alleles that influence risk factors, and the participating genes are “randomized” to determine whether carriers of these genetic variants have a different risk of developing the disease compared to non-carriers (13–15). In addition, using large-scale publicly available data from genome-wide association studies (GWAS) and GWAS meta-analyses to investigate causal associations between exposures and outcomes can significantly enhance the interpretability and reliability of study results (16, 17).

This study aims to investigate the causal relationship between inflammatory cytokines and OLP using bidirectional MR analysis to further explore the pathogenesis of OLP and the effects of OLP on the immune system of the body, and to provide new solutions for the prevention and treatment of OLP.

## Methods

### Study design

**Figure 1:**
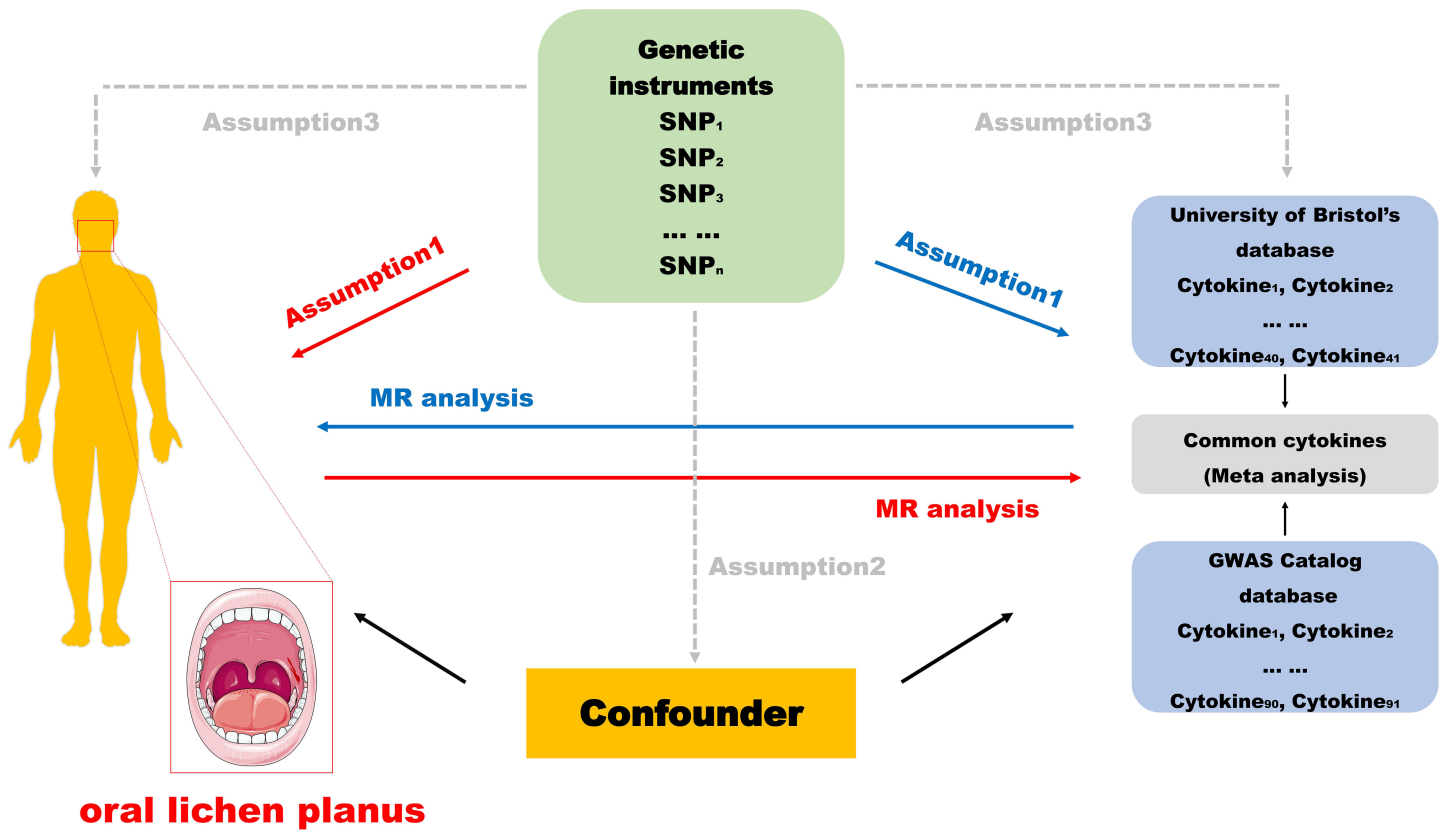
Flowchart of the design.

To visualize the design of this study more, we drew the flow chart (**Figure 1**). In this study, the two-way MR analysis was used. In the forward analysis, we used 132 inflammatory cytokines from two databases as exposures and OLP as the outcome to explore the possibility that different inflammatory cytokines cause OLP. We evaluated the causal relationship between OLP and each inflammatory cytokine in the reverse analysis. The MR analysis was performed using IVs to infer the causal relationship between the exposures and the outcome, and the selected IVs were required to fulfill the following three key assumptions: 1) IVs were significantly associated with exposure; 2) IVs were not associated with any confounders; 3) IVs were not directly associated with outcomes and influenced outcomes only through exposure (15, 18). This study was based on the public GWAS database, and all original studies were ethically approved, requiring no additional informed consent or ethical approval.

### Participants and data sources

Inflammatory cytokine data used in this study were obtained from the GWAS data from the University of Bristol (https://data.bris.ac.uk/data/dataset), and the GWAS Catalog database (ID: GCST90274758-GCST90274848), with each inflammatory cytokine data details are summarized in tabular form (**Supplementary Table 1**) (19). OLP data were obtained from the Finngen database (https://risteys.finregistry.fi/), which includes 6411 case samples and 405770 control samples containing 21306348 SNPs, and the populations included were all European (20).

### Selection of IVs

We screened significantly related SNPs (p<1×10^-5^). To eliminate possible linkage disequilibrium (LD), the significantly related SNPs we screened should satisfy both r^2^<0.01, and KB>500. In addition, palindromic SNPs containing ambiguous linkages should also be corrected or excluded (21–24). We uploaded the screened SNPs to the PhenoScanner website (http://www.phenoscanner.medschl.cam.ac.uk/) to eliminate confounders associated with the outcome. If no SNP matched the outcome pooled data, SNPs significantly associated with the variant (r^2^>0.8) were selected. Data that could not be analyzed subsequently because of missing or insufficient SNPs should be excluded if no alternative SNPs could be found (25, 26). Finally, to exclude potentially weak IVs, we used F>10 as a screening condition to exclude ineligible SNPs. The F value was calculated as F=R^2^(n-k-1)/k(1-R^2^), where R^2^ denotes the extent to which the IVs explain the exposure factors, n denotes the sample size of the exposed GWAS data, and k denotes the number of selected IVs (17, 27, 28).

### MR analysis

In this study, R (4.3.0) was used, and the analysis was based on the “TwoSampleMR” package, the “VariantAnnotation” package, the “gwasglue” package, and the “MRPRESSO” package. The “MRPRESSO” package can be used to determine the robustness of the results and analyze them for heterogeneity (29–31). We used the inverse variance weighted (IVW), MR-Egger, weighted median, simple mode, and weighted mode methods for our analyses, with the IVW method as the primary method for assessing the causal relationship between inflammatory cytokines and OLP because it is based on the random assignment nature of the method, which allows us to mimic a randomized controlled trial and eliminates the problem of endogeneity. In addition, the IVW method improves estimation accuracy by adjusting the weights. The IVW method does not need to satisfy the weak IVs assumption, so more robust estimation results can be obtained. Although the MR-Egger method, simple mode method, weighted mode method and weighted median method are less efficient in analysis, we utilize these methods for **Supplementary Analysis** (32, 33).

We presented the results using the corresponding ratio of OR and 95%CI to visualize the relationship between each inflammatory cytokine and the risk of developing OLP. We tested the results for heterogeneity; we used the Cochran Q statistic, and the results were considered not heterogeneous when p>0.05. Afterward, we calculated the MR-Egger intercept to assess horizontal multivariate validity. In addition, the leave-one-out method was also used for sensitivity analysis to assess the bias that specific SNPs produce on the results (34–36). Finally, we evaluated the same inflammatory cytokine results from both databases after screening based on the “meta” package to confirm the reliability of the positive results. When I^2^<40%, the heterogeneity of results was not significant, while when I^2^>75%, the heterogeneity of results was significant. A random effects model was used when the heterogeneity of results was significant (I^^2^≥50%, or p<0.05), and a common effect model was used when it was not significant (I^^2^<50% and p≥0.05) (24, 37, 38). Since two-way MR analysis was performed between each cytokine and OLP in this study, we used Bonferroni Hochberg correction, i.e., when p<0.00038 (0.05/132), it suggested that the analysis results were significant. However, because multiple analyses lead to very small p-values after correction, some results that might otherwise be associated are often overlooked, which we call suggestive association results, also known as potential positive results, i.e., results that were significant before Bonferroni correction (p<0.05) but not after correction (p>0.00038), and these results were also included in our analyses (39–41).

## Results

### The causal relationship between inflammatory cytokines and OLP

We screened 132 independent SNPs corresponding to OLP-related inflammatory cytokines by filtering against the p-value threshold (p<1×10^-5^). After LD filtering of the remaining SNPs, as well as elimination of SNPs with possible confounders and exclusion of SNPs with F<10, we obtained the final SNPs for subsequent MR analysis.

**Figure 2:**
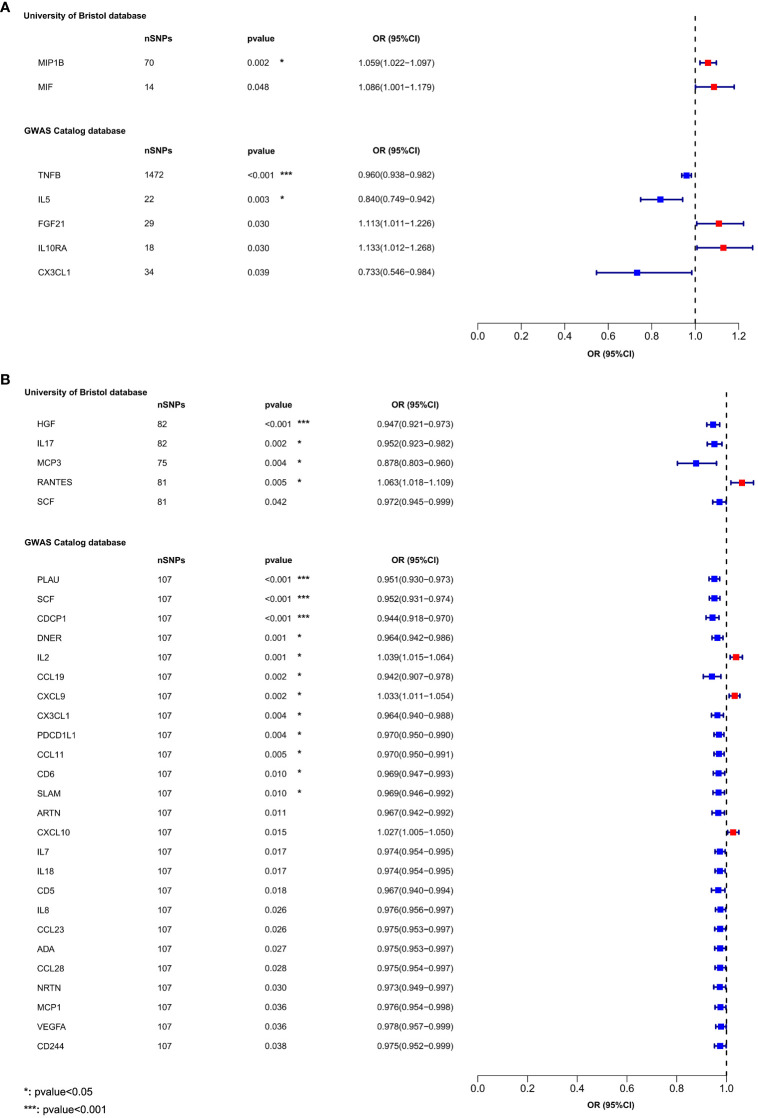
The forest plot shows the causal associations between 132 inflammatory cytokines and OLP, we mainly used the IVW method. OR<1 indicates a negative association between exposure and outcome, while OR>1 indicates a positive association between exposure and outcome. **(A)** Inflammatory cytokines on OLP; **(B)** OLP on inflammatory cytokines.

In the MR analysis of inflammatory cytokines on OLP, we mainly used the IVW method to analyze, and we plotted a forest plot (**Figure 2**) to visualize the results more. We found a total of 7 inflammatory cytokines with a causal relationship with OLP (**Table 1**), among which there was a negative correlation between TNFB, IL5 and CX3CL1 with OLP, with the most significant result being TNFB (p=0.00036, odds ratio (OR): 0.960, 95% confidence interval (95%CI): 0.938-0.982). And the MR-Egger method also corroborated a potential causal relationship and showed a common trend (p=0.00427, OR:0.941, 95%CI:0.903-0.981). Moreover, there is a potential causal relationship between IL5, CX3CL1 and OLP. Similarly, we found potential positive correlations between MIP1B, MIF, FGF21 and IL10RA with OLP, with the most significant relationship between MIP1B and OLP (p=0.00172, OR:1.059, 95%CI:1.022-1.097). In addition to the IVW method, the MR-Egger method also showed a potential association and had the same trend as the IVW method (p=0.00609, OR:1.090, 95%CI:1.027-1.158).

**Table 1:** MR analysis results of inflammatory cytokines on OLP.

University of Bristol database					
Genes	Analysis methods (p-value)				
	IVW	MR Egger	Weighted median	Weighted mode	Simple mode
**MIP1b**	0.00172	0.00609	0.24856	0.24214	0.40905
**MIF**	0.04805	0.39523	0.17493	0.53127	0.50109
GWAS Catalog database					
Genes	Analysis methods (p-value)				
	IVW	MR Egger	Weighted median	Weighted mode	Simple mode
**TNFB**	0.00036	0.00427	0.41014	0.07794	0.74352
**IL5**	0.00288	0.20258	0.13130	0.58227	0.43252
**FGF21**	0.02955	0.17239	0.04953	0.04938	0.21387
**IL10RA**	0.02982	0.85678	0.20496	0.29205	0.09731
**CX3CL1**	0.03894	0.58751	0.35809	0.39880	0.52904

We found that OLP did not increase the expression levels of all inflammatory cytokines during pathogenesis, but that many inflammatory cytokines had decreased in expression levels. In total, we found associations between OLP and the results of 30 inflammatory cytokines (**Table 2**), with negative correlations between HGF, IL17, MCP3, SCF, PLAU, CDCP1, DNER, CCL19, CX3CL1, PDCD1L1, CCL11, CD6, SLAM, ARTN, IL7, IL18, CD5, IL8, CCL23, ADA, CCL28, NRTN, MCP1, VEGFA, CD244 and OLP, with a significant relationship between PLAU, SCF, CDCP1, HGF and OLP (p<0.00038). In addition, the MR-Egger method of these 4 inflammatory cytokines results also showed causal relationship with OLP, and the trend of the relationship was the same as that of the IVW method (PLAU: IVW, p=0.00002, OR: 0.951, 95%CI: 0.930-0.973; MR-Egger, p=0.00097, OR: 0.922, 95%CI: 0.880-0.966; SCF (GWAS Catalog database): IVW, p=0.00002, OR: 0.952, 95%CI: 0.931-0.974; MR-Egger, p=0.00284, OR: 0.930, 95%CI: 0.887- 0.974; CDCP1: IVW, p=0.00004, OR: 0.944, 95%CI: 0.918-0.970; MR-Egger, p=0.00026, OR: 0.897, 95%CI: 0.848-0.949; HGF: IVW, p=0.00009, OR: 0.947, 95%CI: 0.921-0.973; MR-Egger, p=0.01948, OR: 0.942, 95%CI: 0.896-0.989). Finally, we found a potential positive correlation between IL2, CXCL9, RANTES, CXCL10 and OLP, with the most significant relationship between IL2 and OLP (IVW, p=0.00135, OR: 1.039, 95%CI: 1.015-1.064). Although the MR-Egger method did not find a causal relationship between IL2 and OLP (MR-Egger, p=0.85133, OR: 1.005, 95%CI: 0.957-1.055), this method and the IVW method showed the same trend.

**Table 2:** MR analysis results of OLP on inflammatory cytokines.

University of Bristol database					
Genes	Analysis methods (p-value)				
	IVW	MR Egger	Weighted median	Weighted mode	Simple mode
**HGF**	0.00009	0.01948	0.10008	0.08918	0.64967
**IL17**	0.00177	0.00020	0.00496	0.00153	0.38003
**MCP3**	0.00442	0.02179	0.81204	0.81972	0.75165
**RANTES**	0.00511	0.07337	0.00890	0.00444	0.81417
**SCF**	0.04169	0.04258	0.02164	0.00670	0.15096
GWAS Catalog database					
Genes	Analysis methods (p-value)				
	IVW	MR Egger	Weighted median	Weighted mode	Simple mode
**PLAU**	0.00002	0.00097	0.01923	0.20184	0.75603
**SCF**	0.00002	0.00284	0.00731	0.07756	0.13748
**CDCP1**	0.00004	0.00026	0.00714	0.13261	0.58496
**DNER**	0.00126	0.01107	0.03114	0.22002	0.44244
**IL2**	0.00135	0.85133	0.35481	0.53950	0.59552
**CCL19**	0.00191	0.00145	0.02275	0.03033	0.71983
**CXCL9**	0.00241	0.52009	0.45038	0.74029	0.73551
**CX3CL1**	0.00354	0.00016	0.00120	0.01062	0.09328
**PDCD1L1**	0.00412	0.02723	0.01130	0.10368	0.25593
**CCL11**	0.00486	0.16293	0.07862	0.16936	0.25866
**CD6**	0.00971	0.06405	0.62949	0.55540	0.89397
**SLAM**	0.00996	0.13475	0.29444	0.92257	0.35601
**ARTN**	0.01091	0.02510	0.01799	0.04898	0.14750
**CXCL10**	0.01455	0.86293	0.23498	0.23358	0.80685
**IL7**	0.01655	0.00024	0.01218	0.03838	0.34507
**IL18**	0.01727	0.53111	0.13197	0.91173	0.17206
**CD5**	0.01785	0.02318	0.81038	0.77080	0.78506
**IL8**	0.02560	0.04590	0.83399	0.93153	0.58750
**CCL23**	0.02603	0.70591	0.43624	0.78905	0.80434
**ADA**	0.02676	0.00004	0.00370	0.03296	0.41707
**CCL28**	0.02812	0.00269	0.24713	0.43535	0.79701
**NRTN**	0.03033	0.01621	0.21736	0.30179	0.93850
**MCP1**	0.03585	0.01270	0.05448	0.05241	0.49496
**VEGFA**	0.03593	0.16141	0.99817	0.80706	0.39055
**CD244**	0.03787	0.01221	0.03267	0.08671	0.29180

### Sensitivity analysis

**Figure 3:**
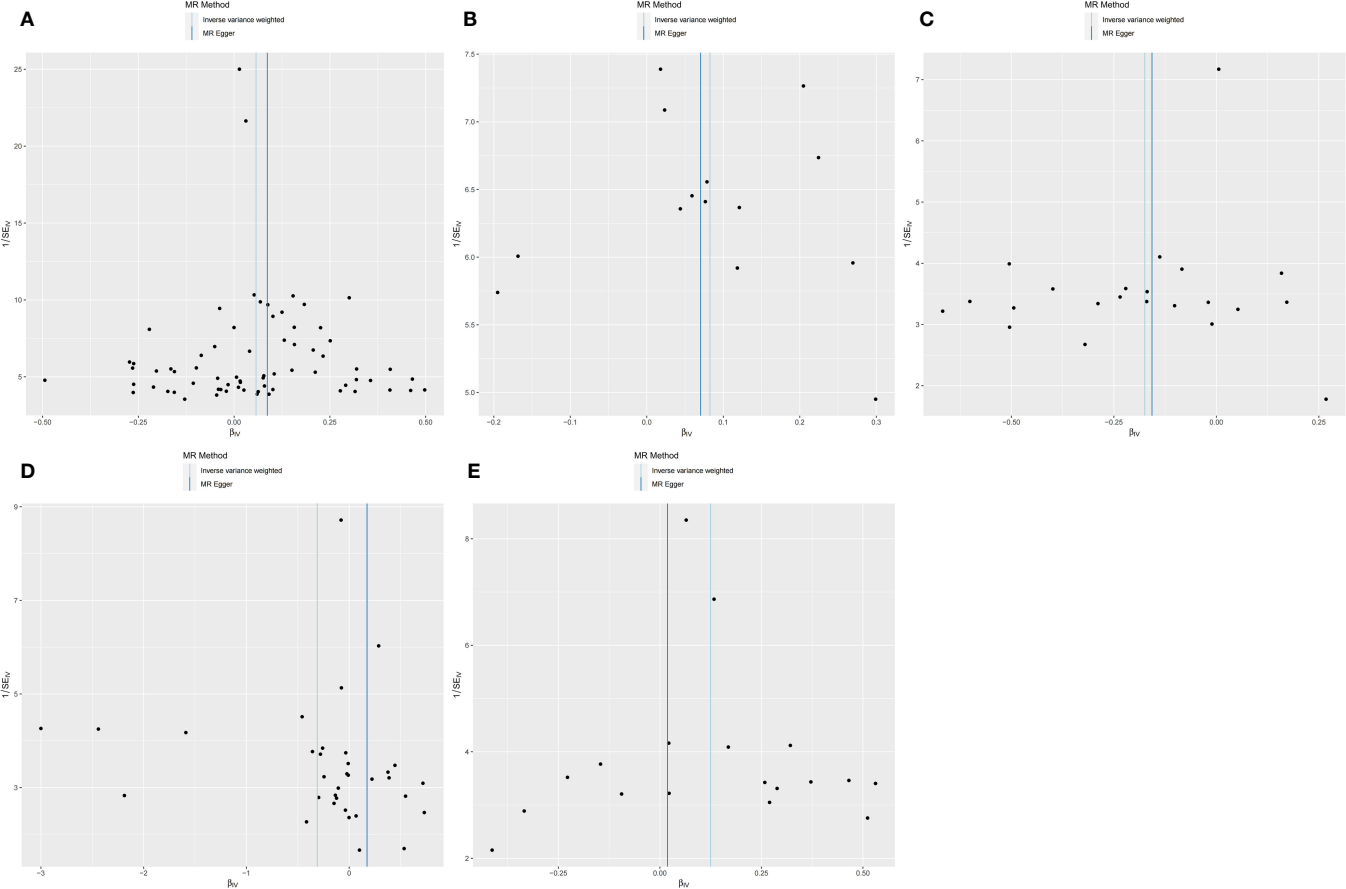
Funnel plot showing heterogeneity analysis of inflammatory cytokines causally associated with OLP. In the analysis of inflammatory cytokines on OLP, we excluded results with heterogeneity and horizontal pleiotropy, and finally screened the results of 5 eligible inflammatory cytokines. **(A)** MIP1B on OLP; **(B)** MIF on OLP; **(C)** IL5 on OLP; **(D)** FGF21 on OLP; **(E)** IL10RA on OLP.

**Figure 4:**
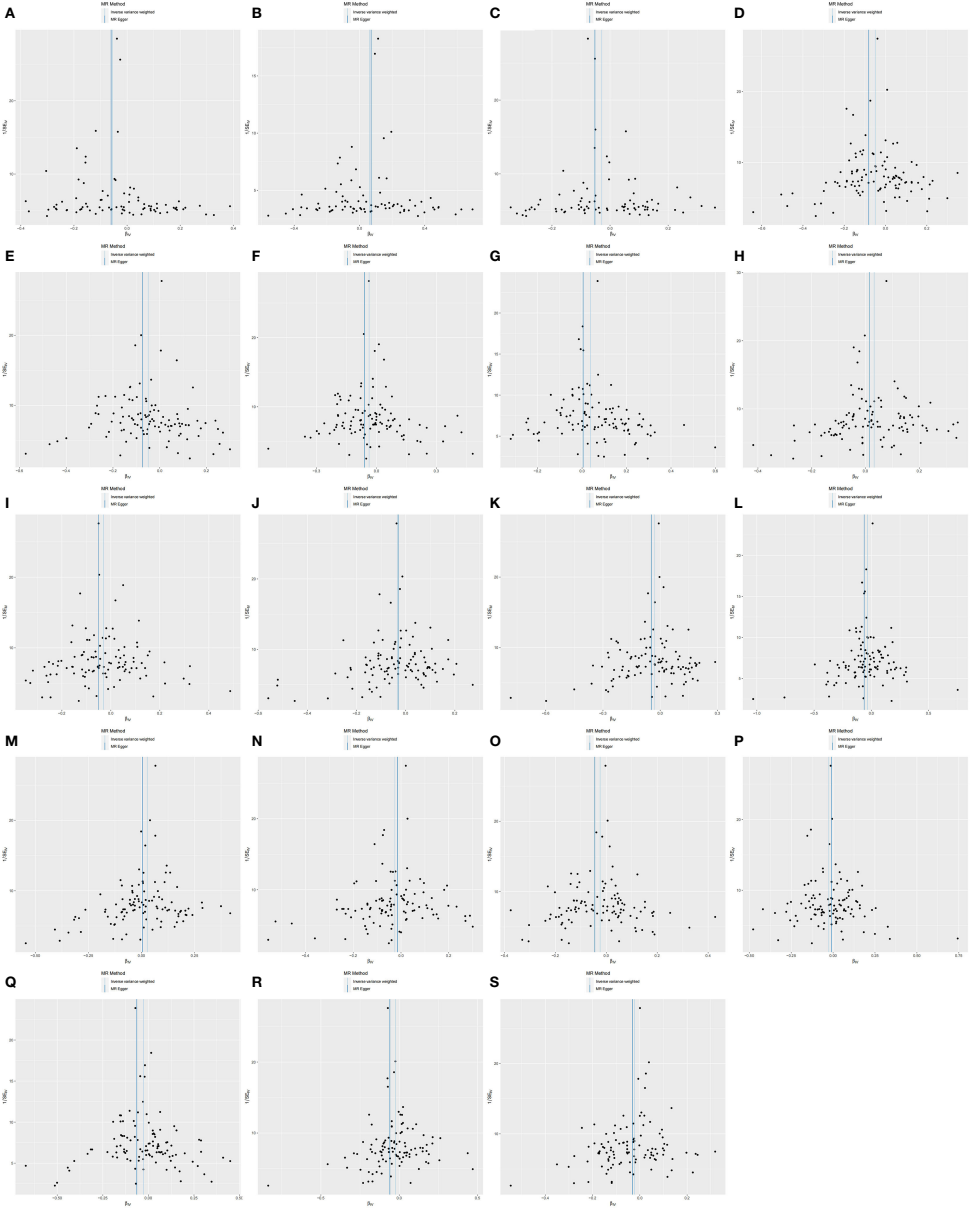
Funnel plot showing heterogeneity analysis of inflammatory cytokines causally associated with OLP. In the analysis of OLP on inflammatory cytokines, we excluded results with heterogeneity and horizontal pleiotropy, and finally screened the results of 19 eligible inflammatory cytokines. **(A)** OLP on HGF; **(B)** OLP on RANTES; **(C)** OLP on SCF (University of Bristol database); **(D)** OLP on PLAU; **(E)** OLP on SCF (GWAS Catalog database); **(F)** OLP on DNER; **(G)** OLP on IL2; **(H)** OLP on CXCL9; **(I)** OLP on PDCD1L1; **(J)** OLP on CCL11; **(K)** OLP on CD6; **(L)** OLP on ARTN; **(M)** OLP on CXCL10; **(N)** OLP on IL18; **(O)** OLP on IL8; **(P)** OLP on CCL23; **(Q)** OLP on NRTN; **(R)** OLP on MCP1; **(S)** OLP on VEGFA.

We performed sensitivity analyses further to validate the causal relationship between inflammatory cytokines and OLP, and the results were summarized in tabular form (**Tables 3**, **4**). We mainly used the IVW method for heterogeneity analysis, supplemented by the MR Egger method for combined analysis. In the heterogeneity analysis of inflammatory cytokines on OLP, we found significant heterogeneity in the results of TNFB and CX3CL1 (TNFB, Qpval=4.29×10^-36^; CX3CL1, Qpval=6.59×10^-53^), so they should be excluded. To present the data intuitively, we visualize the data in the form of a funnel plot (**Figure 3**). After that, we performed the reverse heterogeneity analysis of OLP on inflammatory cytokines. Similarly, we found heterogeneity in the results for MCP3, CDCP1, CCL19, CX3CL1, SLAM, CD5, and CD244 (MCP3, Qpval=0.028; CDCP1, Qpval=5.05×10^-7^; CCL19, Qpval=5.13×10^-25^; CX3CL1, Qpval=0.005; SLAM, Qpval=0.031; CD5, Qpval=1.87×10^-6^; CD244, Qpval=0.027), so these results should be excluded. We visualized the data as the funnel plot (**Figure 4**).

**Table 3:** Sensitivity analysis results (Cytokine on OLP).

University of Bristol database						
Genes	Heterogeneity analysis				Horizontal pleiotropy	
	IVW		MR Egger		Egger intercept	p-value
	Cochran’s Q	Q_pval	Cochran’s Q	Q_pval		
**MIP1b**	83.268	0.116	81.554	0.125	-0.008	0.236
**MIF**	9.496	0.735	9.465	0.663	0.003	0.861
GWAS Catalog database						
Genes	Heterogeneity analysis				Horizontal pleiotropy	
	IVW		MR Egger		Egger intercept	p-value
	Cochran’s Q	Q_pval	Cochran’s Q	Q_pval		
**TNFB**	2255.292	0	2253.466	0	0.002	0.275
**IL5**	15.944	0.773	15.915	0.722	-0.002	0.868
**FGF21**	28.123	0.458	27.848	0.419	-0.006	0.610
**IL10RA**	13.272	0.718	11.701	0.764	0.014	0.228
**CX3CL1**	341.262	0	312.359	0	-0.055	0.095

**Table 4:** Sensitivity analysis results (OLP on Cytokine).

University of Bristol database						
Genes	Heterogeneity analysis				Horizontal pleiotropy	
	IVW		MR Egger		Egger intercept	p-value
	Cochran’s Q	Q_pval	Cochran’s Q	Q_pval		
**HGF**	78.698	0.552	78.633	0.522	0.001	0.799
**IL17**	94.790	0.140	87.769	0.259	0.012	0.013
**MCP3**	98.903	0.028	97.772	0.028	0.013	0.361
**RANTES**	72.343	0.717	72.248	0.691	-0.002	0.758
**SCF**	66.205	0.866	64.956	0.872	0.005	0.267
GWAS Catalog database						
Genes	Heterogeneity analysis				Horizontal pleiotropy	
	IVW		MR Egger		Egger intercept	p-value
	Cochran’s Q	Q_pval	Cochran’s Q	Q_pval		
**PLAU**	124.131	0.110	121.556	0.129	0.006	0.139
**SCF**	121.397	0.146	119.936	0.151	0.004	0.261
**CDCP1**	193.014	0	185.836	0	0.009	0.047
**DNER**	124.816	0.102	123.207	0.108	0.004	0.244
**IL2**	97.161	0.719	94.743	0.754	0.006	0.123
**CCL19**	331.314	0	319.195	0	0.013	0.048
**CXCL9**	107.837	0.432	106.930	0.429	0.003	0.347
**CX3CL1**	146.985	0.005	136.644	0.021	0.011	0.006
**PDCD1L1**	105.125	0.506	104.176	0.504	0.003	0.332
**CCL11**	89.338	0.878	89.336	0.863	0	0.963
**CD6**	129.961	0.057	129.336	0.054	0.003	0.478
**SLAM**	134.877	0.031	134.759	0.027	0.001	0.762
**ARTN**	129.497	0.060	127.783	0.065	0.005	0.238
**CXCL10**	110.267	0.369	108.878	0.378	0.004	0.250
**IL7**	103.515	0.550	94.376	0.762	0.011	0.003
**IL18**	106.317	0.473	105.961	0.455	-0.002	0.554
**CD5**	187.387	0	184.325	0	0.006	0.190
**IL8**	91.095	0.848	89.946	0.852	0.004	0.286
**CCL23**	118.936	0.184	118.222	0.178	-0.003	0.428
**ADA**	122.397	0.132	108.902	0.378	0.013	0
**CCL28**	125.597	0.094	119.701	0.155	0.008	0.025
**NRTN**	117.044	0.218	114.302	0.252	0.007	0.115
**MCP1**	119.564	0.174	116.264	0.213	0.007	0.087
**VEGFA**	89.511	0.875	89.309	0.863	0.002	0.654
**CD244**	135.691	0.027	131.820	0.039	0.007	0.082

**Figure 5:**
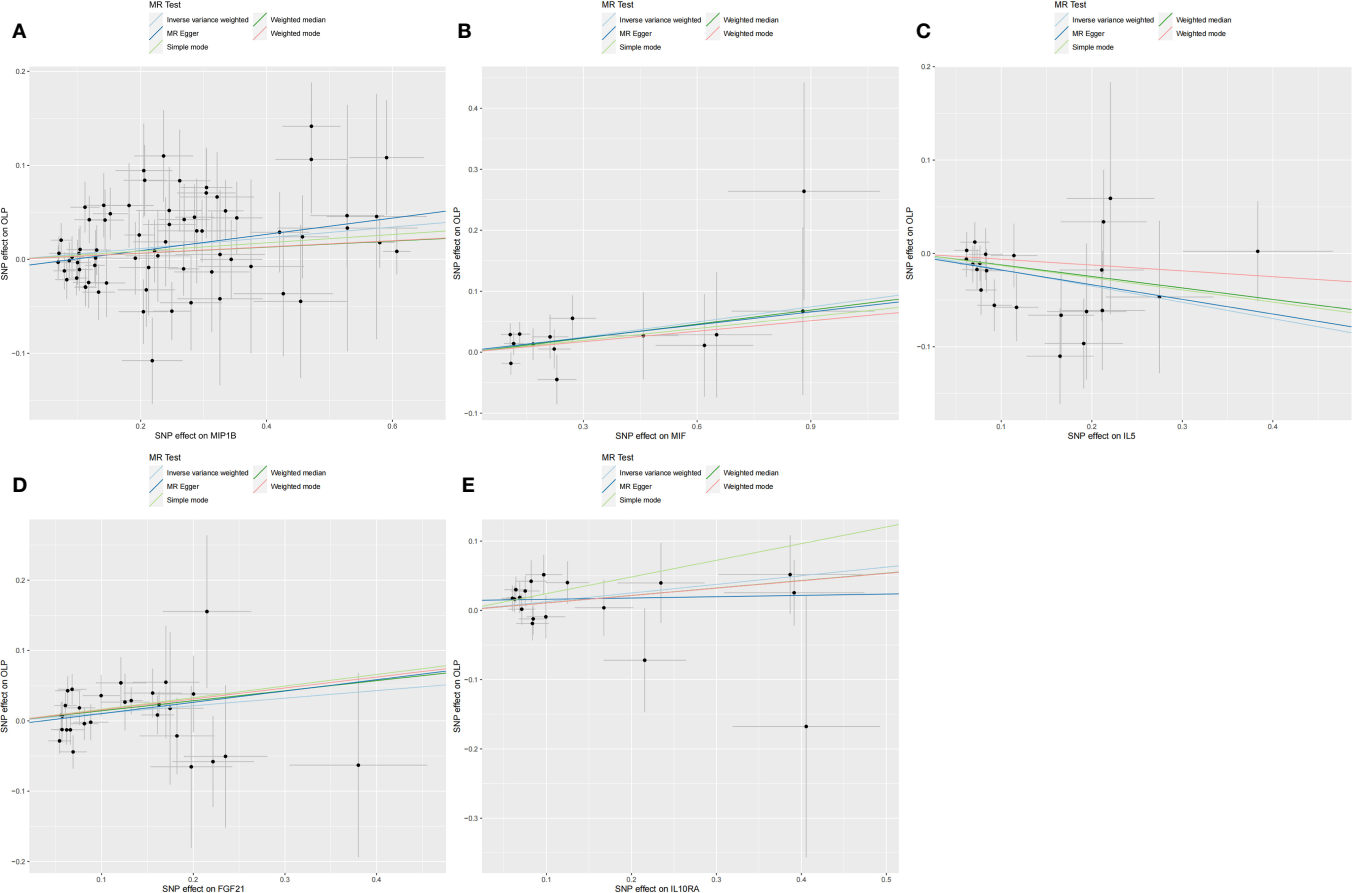
Scatter plot showing the analysis of horizontal pleiotropy of inflammatory cytokines causally associated with OLP. In the analysis of inflammatory cytokines on OLP, we excluded results with heterogeneity and horizontal pleiotropy, and finally screened the results of 5 eligible inflammatory cytokines. **(A)** MIP1B on OLP; **(B)** MIF on OLP; **(C)** IL5 on OLP; **(D)** FGF21 on OLP; **(E)** IL10RA on OLP.

**Figure 6:**
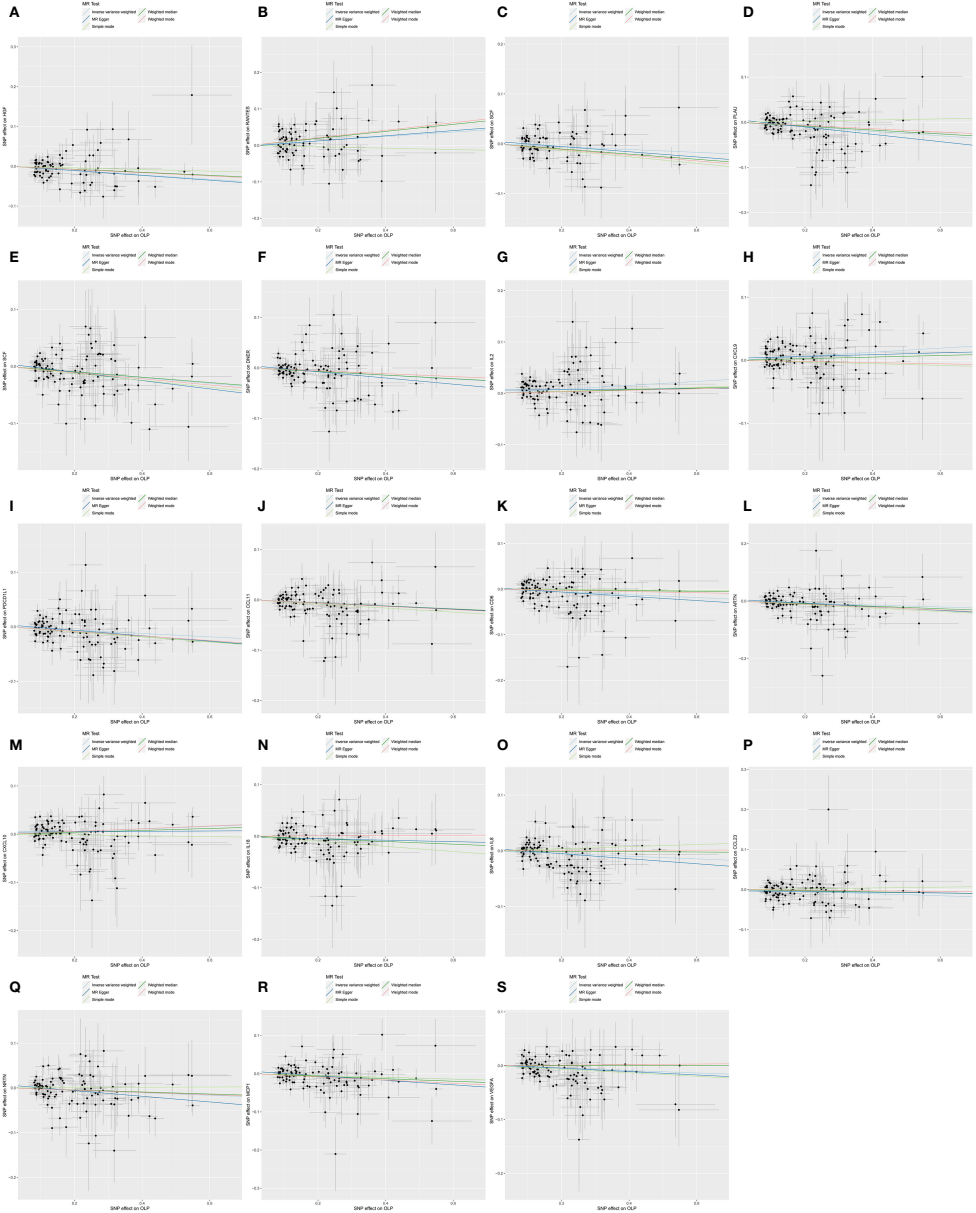
Scatter plot showing the analysis of horizontal pleiotropy of inflammatory cytokines causally associated with OLP. In the analysis of OLP on inflammatory cytokines, we excluded results with heterogeneity and horizontal pleiotropy, and finally screened the results of 19 eligible inflammatory cytokines. **(A)** OLP on HGF; **(B)** OLP on RANTES; **(C)** OLP on SCF (University of Bristol database); **(D)** OLP on PLAU; **(E)** OLP on SCF (GWAS Catalog database); **(F)** OLP on DNER; **(G)** OLP on IL2; **(H)** OLP on CXCL9; **(I)** OLP on PDCD1L1; **(J)** OLP on CCL11; **(K)** OLP on CD6; **(L)** OLP on ARTN; **(M)** OLP on CXCL10; **(N)** OLP on IL18; **(O)** OLP on IL8; **(P)** OLP on CCL23; **(Q)** OLP on NRTN; **(R)** OLP on MCP1; **(S)** OLP on VEGFA.

Subsequently, we performed the validation of horizontal pleiotropy. In performing the validation of horizontal pleiotropy of inflammatory cytokines on OLP, we did not find horizontal pleiotropy between any inflammatory cytokines and OLP, we visualize the data as a scatter plot (**Figure 5**). In performing the test of horizontal pleiotropy of OLP on inflammatory cytokines, we found horizontal pleiotropy between IL17, CDCP1, CCL19, CX3CL1, IL7, ADA, CCL28 and OLP (IL17, pval=0.013; CDCP1, pval=0.047; CCL19, pval=0.048; CX3CL1, pval=0.006; IL7, pval=0.003; ADA, pval=4.76×10^-4^; CCL28, pval=0.025), so these results should be excluded. We visualized the results (**Figure 6**).

**Figure 7:**
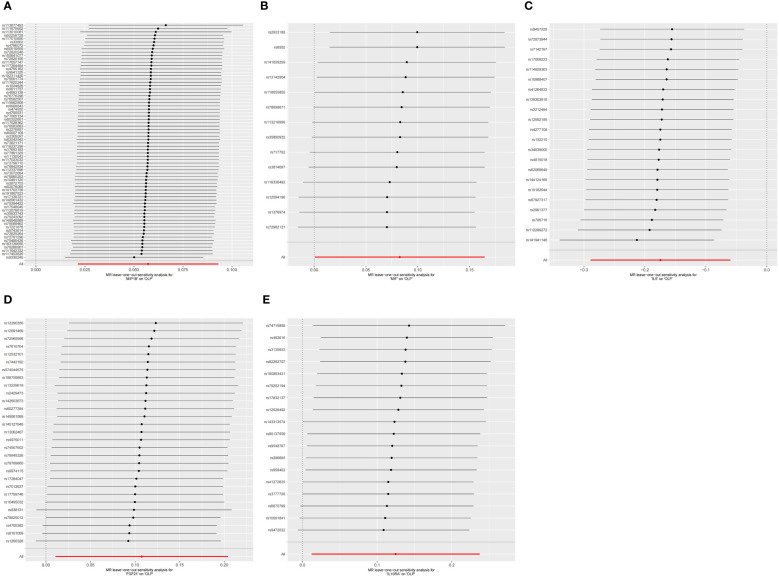
The forest plot shows the results of leave-one-out analyses, where we found no SNPs that could bias the results in the analysis of inflammatory cytokines on OLP. **(A)** MIP1B on OLP; **(B)** MIF on OLP; **(C)** IL5 on OLP; **(D)** FGF21 on OLP; **(E)** IL10RA on OLP.

**Figure 8:**
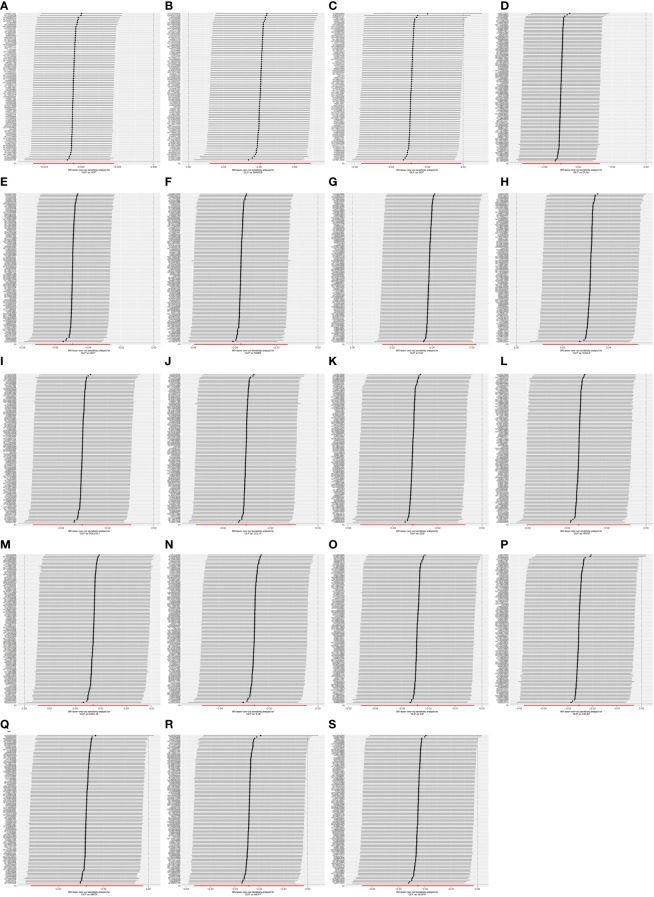
The forest plot shows the results of leave-one-out analyses, where we found no SNPs that could bias the results in the analysis of OLP on inflammatory cytokines. **(A)** OLP on HGF; **(B)** OLP on RANTES; **(C)** OLP on SCF (University of Bristol database); **(D)** OLP on PLAU; **(E)** OLP on SCF (GWAS Catalog database); **(F)** OLP on DNER; **(G)** OLP on IL2; **(H)** OLP on CXCL9; **(I)** OLP on PDCD1L1; **(J)** OLP on CCL11; **(K)** OLP on CD6; **(L)** OLP on ARTN; **(M)** OLP on CXCL10; **(N)** OLP on IL18; **(O)** OLP on IL8; **(P)** OLP on CCL23; **(Q)** OLP on NRTN; **(R)** OLP on MCP1; **(S)** OLP on VEGFA.

Finally, we performed the leave-one-out analysis of the data. In the forward analysis, no specific SNP drove the association between inflammatory cytokines and OLP. The analysis results are presented as the forest plot (**Figure 7**). In the reverse analysis, we did not find any particular SNP significantly affected the results, and we visualized the results (**Figure 8**).

### Meta-analysis

**Figure 9:**
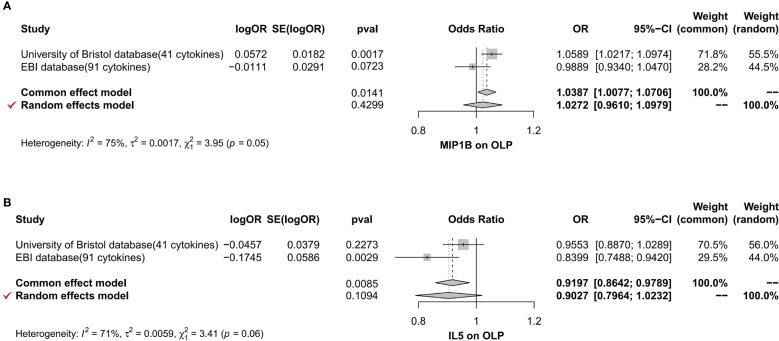
The forest plot shows the two sets of results from the analysis of inflammatory cytokines on OLP by meta-analysis methods combined to assess the reliability of positive or potentially positive results, and we mainly analyzed the results of the IVW method and the selection of effect models. The forest plot uses the effect point as the invalid line when it equals 1. When 95% of the effect size contains 1 or is equal to 1, i.e., when the diamond-shaped region in the forest plot intersects with the invalid line, it suggests that the combined results are not statistically significant. When the diamond-shaped region invalid line does not cross, it means that the mixed results are statistically significant. **(A)** The meta-analysis of MIP1B; **(B)** The meta-analysis of IL5.

**Figure 10:**
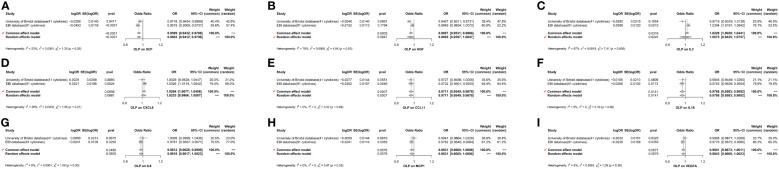
The forest plot shows the two sets of results from the analysis of OLP on inflammatory cytokines by meta-analysis methods combined to assess the reliability of positive or potentially positive results. **(A)** The meta-analysis of SCF; **(B)** The meta-analysis of HGF; **(C)** The meta-analysis of IL2; **(D)** The meta-analysis of CXCL9; **(E)** The meta-analysis of CCL11; **(F)** The meta-analysis of IL18; **(G)** The meta-analysis of IL8; **(H)** The meta-analysis of MCP1; **(I)** The meta-analysis of VEGFA.

To further reduce the bias of results due to different data sources, we performed a meta-analysis of the results of the same inflammatory cytokines from different database sources. In the forward analysis, we finally screened 3 inflammatory cytokines, namely FGF21 (p=0.02955, OR: 1.113, 95%CI: 1.011-1.226), IL10RA (p=0.02982, OR: 1.133, 95%CI: 1.012-1.268) and MIF (p=0.04805, OR: 1.086, 95%CI: 1.001-1.179). We visualize the results in the form of a forest plot (**Figure 9**). In the reverse analysis, we screened 14 inflammatory cytokines, namely PLAU (p=0.00002, OR: 0.951, 95%CI: 0.930-0.973), SCF (University of Bristol database, p=0.04169, OR: 0.972, 95%CI: 0.945-0.999; GWAS Catalog database, p=0.00002, OR: 0.952, 95%CI: 0.931-0.974), DNER (p=0.00126, OR: 0.964, 95%CI: 0.942-0.986), CXCL9 (p= 0.00241, OR: 1.033, 95%CI: 1.011-1.054), PDCD1L1 (p=0.00412, OR: 0.970, 95%CI: 0.950-0.990), CCL11 (p=0.00486, OR: 0.970, 95%CI: 0.950-0.991), RANTES (p=0.00002, OR: 0.952, 95%CI: 0.931-0.974), CD6 (p=0.00971, OR: 0.969, 95%CI: 0.947-0.993), ARTN (p=0.01091, OR: 0.967, 95%CI: 0.947-0.993), CXCL10 (p= 0.01455, OR: 1.027, 95%CI: 1.005-1.050), IL18 (p=0.01727, OR: 0.974, 95%CI: 0.954-0.995), IL8 (p= 0.02560, OR: 0.976, 95%CI: 0.956-0.995), CCL23 (p=0.02603, OR: 0.975, 95%CI: 0.953-0.997) and NRTN (p=0.03033, OR: 0.973, 95%CI: 0.949-0.997). We found, among them, a significant causal relationship between PLAU, SCF and OLP (p<0.00038), and a trend toward negative correlation with OLP. We visualized the final results (**Figure 10**).

## Discussion

This study more comprehensively revealed the causal relationship between inflammatory cytokines and OLP using bidirectional Mendelian randomization analysis. The forward analysis showed that among the 132 inflammatory cytokines results, 3 inflammatory cytokines and OLP had a causal relationship, and all 3 inflammatory cytokines were positively correlated with OLP. However, it is noteworthy that among the 14 inflammatory cytokines screened by reverse analysis, we found a negative correlation between most of the inflammatory cytokines and OLP.

Many studies have shown that cytokines play a key role in the pathogenesis of OLP. Th1 and Th2 cells belong to the CD4+ T-cell subpopulation, and the chronic migratory damage characteristic of OLP is closely related to these two cells. The equilibrium between Th1 and Th2 cells is the key factor in maintaining the stability of the immune system, while Th1 and Th2 cells can regulate each other through secreting cytokines and other cells. If this equilibrium is disrupted, immune cell drift dominated by Th1 or Th2 cells occurs in the body, in which case there is a significant increase in the incidence of immune diseases (42, 43). Some studies have shown that Th1 cells can produce immune effects by secreting a large number of inflammatory cytokines (e.g., IL2, IFN-γ) etc., and the produced inflammatory cytokines, such as IL2, etc., can further promote the differentiation of Th0 cells to Th1 cells and increase the number of Th1 cells, which will lead to an imbalance of Th1/Th2 (2, 44, 45). However, the immune imbalance state of the organism also involves the participation of Th2 cells, and the cytokines produced by Th2 cells (e.g., IL4, IL10) can inhibit the function of Th1 cells, thus regulating the immune imbalance state. Furthermore, the energy metabolism and cellular function of macrophages in the OLP lesion area are also in an active state, and some reports have shown that they release a variety of biologically active substances during their activation, which can aggravate the damage to local tissues in OLP lesions (6, 8, 46).

Many previous studies have found that the expression levels of some inflammatory cytokines can dominate the pathogenesis and prognosis of ADs. Malignant changes in ADs are often accompanied by sudden increases or decreases in the expression levels of inflammatory cytokines. This makes cytokine screening and changes in expression levels the focus of our studies. By screening the data of 132 cytokines in this study, we found that aberrant expression of FGF21, IL10RA and MIF may increase the risk of OLP. FGF21 belongs to the isoform of the fibroblast growth factor family FGF19, a secreted protein that regulates the body’s metabolism. Currently, FGF21 has been widely used in the prevention and rehabilitation of metabolic diseases such as hepatic lipid and glycolipid metabolism as well as cardiovascular diseases, and it may become one of the effective targets for metabolic disease prevention and rehabilitation (47). However, previous studies have reported the relationship between FGF21 and ADs. Hulejová et al. found that (48) the expression level of FGF21 in serum and synovial fluid of rheumatoid arthritis (RA) patients was significantly higher than that of controls, and the expression level was positively correlated with the body mass index (BMI) of the patients. However, the exact mechanism of FGF21’s role in the pathogenesis of RA is unknown, and the authors suggest that it may be related to an increase in the compensatory response in patients. IL10 plays an important role in B-cell activation and autoantibody production. Still, the pleiotropic properties of IL10 are achieved by binding to the IL10 receptor, in which IL10RA plays a role that cannot be ignored. A study of IL10RA and RA showed that (49) the frequency of certain alleles of IL10RA was significantly higher than that of controls. Among several single nucleotide polymorphic loci selected by the authors, abnormalities in rs9610 can significantly increase susceptibility to RA. MIF can be produced by activated T cells (Th1 cells), B lymphocytes, or macrophages, and this cytokine inhibits the anti-inflammatory effects of glucocorticoids and regulates macrophage function in host defense species. Although the role of MIF in OLP is unclear, several studies have found that MIF is more actively expressed in some ADs. One study found that (50) circulating MIF levels were significantly elevated in RA patients, and an association between MIF-173 C/G and MIF-794CATT5-8 and susceptibility to RA was identified. In addition, a significant increase in MIF levels was observed in the cerebrospinal fluid of patients with relapsing-remitting multiple sclerosis (RRMS), and circulating MIF levels were higher in patients with progressing disability than those with stable disease (51, 52). The above findings suggest that our findings generally agree with previous studies that some inflammatory cytokines with significantly elevated expression levels have a role in exacerbating ADs.

However, by reverse Mendelian randomization analysis, we found a negative correlation between most inflammatory cytokines and OLP, implying that as the disease course of OLP progresses, it is often accompanied by a decrease in the expression levels of some cytokines. Among the 14 cytokines screened, PLAU and SCF showed the most significant causal relationship with OLP (p<0.00038), and it is noteworthy that SCF data from 2 databases both. PLAU, also known as urokinase-type plasminogen activator (uPA), is an essential serine protease that plays a variety of roles in the human body, primarily involved in cell proliferation, migration, and invasion, as well as regulation of fibrinogen activation. There are fewer reports on PLAU and ADs, but one study still report a relationship between the two. In the study on the characterization of nephritis in a mouse model of ADs (53), it was found that in diseased mice, there was often an overexpression of plasminogen activator inhibitor 1 (PAI-1), which led to an increase in the expression of procoagulant molecule tissue factor (TF) and decreased expression of uPA, which leads to the formation of microthrombi and promotes the progression of lupus nephritis (LN). However, different results have been given in reports of other ADs. In a study by Muzio et al. (54), evidence was found for high expression of PLAU in spiny loose cells from patients with pemphigus vulgaris (PV), and the authors concluded that this phenomenon was likely related to an abnormal distribution of albumin. SCF is an important hematopoietic growth factor that acts mainly on the early pluripotent stem and progenitor cells and late-mature blood cells. Some studies have also now found that SCF plays a role in some ADs. In a study by Jin et al. (55), it was found that intraperitoneal injection of SCF into an experimental autoimmune encephalomyelitis (EAE) mouse model effectively restored the number of interstitial cells of Cajal (ICC), which improved bladder dysfunction due to EAE in mice. This also suggests that the symptoms of EAE can be effectively improved by regulating the level of SCF. Furthermore, a study by Massolt et al. showed that (56) serum SCF secretion levels were lower in a susceptible population to autoimmune thyroid disease (AITD) with negative TPO-Ab, i.e., the non-seroconverting (NSC) population than in healthy controls; however, when this population develops seroconverting (SC), serum SCF levels were significantly higher than in healthy controls. Although the authors did not explicitly suggest a reason for this paradigm shift, they hypothesized from the expression status of neural stem cells that this shift is likely to result from the immunosuppression process to the full onset of an immune response. In general, we believe that the relationship between the immune system and ADs is extremely complex, and that the response patterns and expression levels of some inflammatory cytokines may vary at different stages of ADs, which suggests that we should pay attention to the different effects of disease progression on the expression of cytokines in related studies.

Although we found only a few causal relationships between cytokines and OLP among the 132 cytokines, and most had only potential causal relationships with OLP, this does not entirely imply that the remaining cytokines are not associated with OLP. In previous studies, we can find that cytokines of the interleukin family are closely related to the development of OLP, such as IL1, 2, 4, 5, 6, 8, 10, 12, 17, 18, etc., all of which play an indispensable role in the pathogenetic process of OLP (4, 6). IL2 is a marker for T lymphocyte activation, and high levels of IL2 expression can usually be found in the lesion area of OLP, while IL2 can play a regulatory role in the expansion and activation of infiltrating T lymphocytes in the lesion area (45, 57). As for IL4, which is mainly produced by Th2 cells, some studies have found that the expression of IL4 levels in OLP lesions is significantly elevated, and it is believed that there is some correlation with the susceptibility to OLP (46). The relationship between IL6 and OLP has also been studied in depth up to now, and large amounts of IL6 can be found widely in the lesions, peripheral blood, and saliva of patients with OLP, and is more pronounced in erosive OLP (EOLP). There is a close association between IL6 and invasive lymphocytes and keratinocytes, and the presence of large amounts of IL6 can increase pro-inflammatory factors and enhance the local inflammation in OLP lesions (58). In addition, studies on the role of other interleukin family cytokines in the pathogenesis of OLP have been reported accordingly (6). Cytokines such as TNF-α and IFN-γ also play an important role in the development of OLP (45, 59, 60). Based on the above research reports, we can find that the relationship between inflammatory cytokines and OLP is complex, and it is difficult to fully sort out the real vein between the two through previous single experiments.

MR analysis is a data analysis method for assessing etiological inferences in epidemiological studies that utilize genetic variants with strong correlations with exposure factors as IVs to assess causal relationships between exposures and outcomes. At its core, it utilizes Mendel’s second law, i.e., the law of independent assortment, which has advantages not found in many experimental research methods, as genetic variants are not subject to confounding factors, such as social behavior and psychology, and can well reduce possible bias in the results. Moreover, MR studies are generally analyzed based on data from large-scale experimental samples, which is more reliable than general experimental studies (13, 15, 61). In this study, we found strong evidence that inflammatory cytokines affect the risk of OLP development, which provides a reliable research idea for further understanding the pathogenesis of OLP.

This study used GWAS public data to incorporate a large number of experimental samples with genetic information, all of which originated from a single population, avoiding biased results due to population differences. However, we must also recognize some limitations of the MR study. This study is based on the principle of the three main assumptions, which is limited by the limitations of MR analysis, and the reliability of the second and third assumption cannot be verified perfectly. Our results found horizontal pleiotropy in IL17, CDCP1, CCL19, CX3CL1, IL7, ADA, and CCL28, suggesting that confounding factors affecting outcome still exist in some single SNPs. Moreover, in the heterogeneity analysis, we also found the existence of heterogeneity for TNFB, CX3CL1, MCP3, CDCP1, CCL19, SLAM, CD5 and CD244. In a meta-analysis of the results of the same cytokines from different database sources, we excluded the results of MIP1B, IL5, HGF, IL2, MCP1, and VEGFA, which were not statistically significant after merging. The heterogeneity of a larger number of results may be due to different data sources on the one hand. And on the other hand, we still cannot completely exclude some potential confounders. For example, the lack of detailed subject information prevented us from proceeding to subgroup analysis, resulting in biased results. The population sources used in this study are all from European populations. If the results are directly applied to other races, ethnic bias may occur, so we need to further confirm the results of this study with more research samples.

## Conclusion

In summary, the present study found evidence that inflammatory cytokines play a crucial role in OLP, and it provides a reliable analytical idea for the subsequent research of targeted immunomodulation in patients with OLP. Still, this idea needs to be validated by further clinical and basic experiments.

## Data availability statement

The original contributions presented in the study are included in the article/**Supplementary Material**. Further inquiries can be directed to the corresponding author.

## Ethics statement

Ethical approval was not required for the study involving humans in accordance with the local legislation and institutional requirements. Written informed consent to participate in this study was not required from the participants or the participants’ legal guardians/next of kin in accordance with the national legislation and the institutional requirements.

## Author contributions

XC: Validation, Visualization, Writing – original draft. SZ: Visualization, Writing – original draft. XW: Visualization, Writing – original draft. YL: Writing – review & editing. BL: Writing – review & editing. ZZ: Investigation, Resources, Writing – review & editing.

## Supplementary material

Supplementary Table 1 Data sources and information for the MR study.

Supplementary Table 2 Results of the analysis of inflammatory cytokines derived from the University of Bristol database (Inflammatory cytokines on OLP).

Supplementary Table 3 Results of the analysis of inflammatory cytokines derived from the GWAS Catalog database (Inflammatory cytokines on OLP).

Supplementary Table 4 Results of the analysis of inflammatory cytokines derived from the University of Bristol database (OLP on inflammatory cytokines).

Supplementary Table 5 Results of the analysis of inflammatory cytokines derived from the GWAS Catalog database (OLP on inflammatory cytokines).
